# Abstinence. Professor Hitchcock

**Published:** 1831

**Authors:** 


### LXXII.

### Abstinence.—Professor Hitchcock.

In a little work, entitled "Dyspepsy forestalled and resisted," published in America, by Professor Hitchcock, of Amherst College, we have a more amusing and instructive collection of peptic precepts, and sanatory regulations than in any other work which has fallen into our hands. We are only able, in this Number of the Journal, to announce the book, and make a single extract at random. On future occasions we shall glance at several subjects discussed by the American Professor.

### Abstinence.

"To those literary men who are determined in spite of arguments and physicians, to live as they have lived, in the indulgence of their appetites, there is one direction, which may serve to obviate the effects of their excesses. It is the observance of occasional seasons of abstinence from food. This is the sovereign remedy the brutes employ for nearly all their diseases; and, indeed, in chronic complaints, it has a wonderful power over the human constitution. John Home, the author of Douglas, and otherwise distinguished in literature, bore without inconvenience the luxuries of London, by eating on the Sabbath nothing more than a single poached egg.* This course was not only wise, but Sunday was the best day of the seven for observing such a fast.

EXAMPLES OF ABSTINENCE:—HOWARD, FRANKLIN, SOCRATES, &C.

A French writer, in a work entitled 'An Apology for Fasting,' has made a comparison between the longevity of 152 bishops or clergymen, who not only led strictly temperate lives, but frequently fasted, and the same number of men devoted to literature and science, who were also temperate but not abstinent; and he finds that the lives of the bishops were seven years longer, upon an average, than those of the Academicians; which he imputes to the effects of fasting.

Howard, the philanthropist, fasted one day in the week; as did also Franklin, for a time: and Bonaparte, when he felt his system unstrung, gave up his usual repasts, and took exercise on horseback.†

The ablest physicians declare that abstinence is one of the most effectual means of preventing violent and inflammatory disorders, such as fevers and sore throats. Socrates lived in Athens during the whole of the celebrated plague, that made such desolation in that city in his time; yet he escaped unhurt; and this is unanimously ascribed, by the writers of those times, to his uninterrupted temperance.‡

I do not mean by abstinence in this case, that which is excessive: not such as monkish austerity has so often practised, and which has been the source of so much religious superstition, self-righteousness, and cruelty. Seasons of entire abstinence, unless directed by a physician, are rarely extended with advantage, beyond a day or two. But the danger on this side is so small, that a caution seems at this day scarcely necessary.

CASE OF POMPONIUS ATTICUS.

An amusing and instructive example of the good effects of abstinence, occurs in the case of Pomponius Atticus, the friend of Cicero. The melancholy accompanying a disordered stomach, brought him to the resolution of destroying himself; and he called together his friends, to consult as to the best means of accomplishing his design. His son-in-law, Agrippa, with great sagacity, advised and persuaded him to starve himself; recommending, however, that he should occasionally swallow a little water, to alleviate the pains of abstinence. To this he consented, supposing, as people generally do at this day, that water affords no nourishment, although, in fact, it is almost the only fluid that does nourish. In this case it sustained Atticus, day after day, beyond the time he had calculated upon dying by starvation: and not only so, but this water and the abstinence, removed the complaints of his stomach, and his dejection of spirits; and he was then easily persuaded by his son, that it was his duty to live. He did live to an advanced age."

* "Sure Methods, &c. p. 88."

† "Journal of Health, Vol. I. p. 13."

‡ "Spectator, 165, also Rees' Cyc. Art. Abstinence."

---

LXXIII.

